# Calibration Strategies for Correcting Matrix Effects in ICP-MS Multi-Element Analysis Using a Certified Reference Material Followed by Application to Brown Crab (*Cancer Pagurus*) Tissues

**DOI:** 10.3390/foods15213845

**Published:** 2026-10-27

**Authors:** Dongli Dong, Raquel Cama-Moncunill, David van Acken, Selene Pedrós-Garrido, Daniel Hurley, James G. Lyng

**Affiliations:** 1 UCD School of Agriculture and Food Science, University College Dublin, D04 V1W8 Dublin, Ireland; dongli.dong@ucdconnect.ie (D.D.); raquel.cama-moncunill@ucd.ie (R.C.-M.); daniel.hurley@ucd.ie (D.H.); 2 UCD School of Earth Sciences, University College Dublin, D04 V1W8 Dublin, Ireland; david.vanacken@ucd.ie

**Keywords:** ICP-MS, multi-element analysis, matrix effects, calibration strategy, standard addition, certified reference material, crab tissues

## Abstract

Accurate multi-element quantification in seafood by inductively coupled plasma mass spectrometry (ICP-MS) can be compromised by matrix effects, particularly when aqueous-based calibration is applied to complex biological digests. This study evaluated five calibration strategies for the determination of As, Cd, Cr, Ni, Pb and Se in seafood matrices using a certified reference material (CRM, ERM^®^-CE278k, mussel tissue) and brown crab (*Cancer pagurus*) hepatopancreas and claw meat. External calibration (EC), EC with internal standard (EC-IS), standard addition (SA), SA with internal standard (SA-IS), and single-level SA-IS (SLSA-IS) were first compared using the CRM. Matrix effects were quantified from the slope ratio between SA and EC calibration curves. Significant signal suppression of −27.75% was observed for Se with the 95% confidence interval (CI) from −41.2% to −14.3% and −18.1% for Cd (95% CI: −25.2% to −11.0%), while As showed a borderline suppression trend (−15.1 ± 6.2%, *p* = 0.052) with the 95% CI from −30.6 to 0.3%. External calibration and EC-IS gave acceptable precision but underestimated As, Cd and Se, with biases of −25.66%, −22.63% and −35.54% for EC and −20.16%, −17.03% and −31.47% for EC-IS, respectively. Standard addition-based calibration improved trueness, and SA-IS produced the closest agreement with the certified elemental concentration, with biases of −2.91%, 1.20%, −10.34%, 2.92%, −0.30% and −3.42% for As, Cd, Cr, Ni, Pb and Se, respectively. The performance of SLSA-IS was spike-dependent (1–50 µg/L), with higher spike levels yielding the most accurate predictions. Subsequently, SA-IS was explored in hepatopancreas and claw meat from two brown crabs, providing satisfactory determinations for Cd, Cr, Ni, Pb and Se, whereas As quantification was limited by high native concentrations exceeding the standard-addition range in the present study. Cross-matrix evaluation showed that agreement between MMC and SA-IS was element-, matrix- and batch-dependent, with crab tissue-based MMC generally showing closer agreement than CRM-based MMC. Therefore, MMC deserves further investigation for selected applications.

## 1. Introduction

Brown crab (*Cancer pagurus*) is one of the most economically important crustacean species in Europe, appreciated by consumers worldwide. While valued for its nutritional and commercial significance, brown crab also poses food safety concerns. As a carnivorous species with a long life span, brown crab is prone to bioaccumulating both essential and toxic trace elements, such as arsenic, cadmium, chromium, mercury, nickel, lead and selenium (As, Cd, Cr, Hg, Ni, Pb and Se), particularly in metabolically active tissues such as the hepatopancreas and gills [1]. For public health, many countries and regulatory authorities have established maximum limits for most of these elements in crustacean products. For example, the European Union (EU) sets limits of 0.5 mg/kg for Pb, Cd and Hg in crustacean muscle meat [2]. The Australia New Zealand Food Standards Code restricts inorganic As to 2 mg/kg [3].

Depending on the element, the choice of crab tissue for analysis can significantly influence the measured concentration because trace-element accumulation is highly tissue-specific in aquatic animals [1,4,5]. For instance, the study of Cd distribution in crab (*Portunus trituberculatus*) by Zhao et al. [6] has shown that brown meat accumulated higher concentrations of 4.13 ± 2.55 mg/kg (wet weight) than the whole body (2.76 ± 2.34 mg/kg, wet weight) and a much lower concentration of 0.75 ± 0.51 mg/kg in white meat. Regulatory frameworks also reflect tissue considerations. For example, in the EU, the maximum Cd level explicitly applies only to muscle meat from crab appendages (cephalothorax excluded), while China’s GB 2762-2022 sets the limit on the edible portion (viscera are not specifically excluded in practice) [7], and South Korea sets different limits depending on whether viscera are present in the sample [8]. Therefore, the regulatory discrepancy and the anatomical distribution difference of trace elements highlight the need for accurate and tissue-specific quantification methods.

Microwave-assisted acid digestion followed by inductively coupled plasma mass spectrometry (ICP-MS) is widely employed for the determination of trace elements in seafood, due to its high sensitivity and multi-element capability [9,10,11]. However, its analytical performance is often compromised by matrix effects (MEs) defined as the combined effect of all components of the sample other than the analyte on the measurement of the quantity [12,13]. Complex matrices, including digestion reagents and residual carbon, dissolved salts and other tissue-derived components, can affect the sample uptake efficiency to the plasma, plasma ionisation and ion transmission, leading to signal suppression or enhancement and ultimately biased quantification [14]. Dilution of digests is a commonly recommended and straightforward approach to mitigate MEs [13]. However, this approach may not be appropriate when analyte concentration is very low, as dilution may reduce levels to near the limit of quantification (LoQ) or even the limit of detection (LoD) [10,15]. Therefore, calibration strategies capable of compensating for MEs are required.

External calibration (EC) using aqueous standards is simple and widely applied, yet it does not inherently account for biases caused by MEs. Matrix effects can be broadly manifested as proportional effects that alter calibration sensitivity, additive/constant effects that alter the background or intercept, composite effects involving both, and, in more complex cases, non-linear responses. Ideally, using an analyte-free real sample matrix as the calibration solvent will minimise such bias. However, blank crab tissue matrices are difficult to obtain and reproduce. In this case, the standard addition (SA) method is widely used because known amounts of analytes are added to the sample itself, allowing calibration within the sample matrix and effective compensation for proportional MEs [16]. However, additive/constant effects require appropriate background or interference correction, while non-linear effects cannot be reliably compensated by conventional linear SA if the response deviates from linearity over the addition range [16]. The requirement to construct matrix-specific calibration curves makes SA reagent-intensive, labour-intensive and time-consuming. Single-level SA (SLSA), by contrast, is based on paired measurements of spiked and non-spiked sample solutions. On the other hand, because SLSA only depends on a single increment rather than calibration curves, it is highly sensitive to spike design and measurement noise [14,16]. Alternatively, matrix-matched calibration (MMC) aims to reduce MEs by preparing calibration standards in a matrix which mimics the sample matrix as closely as possible. Therefore, developing a calibration using a certified reference material (CRM) with a biological matrix comparable to that of the samples could potentially reduce MEs and reduce extensive sample preparations associated with the SA method [16]. This feature may be advantageous when the aim is to analyse a large number of samples and/or different tissues, which all have similar, though not identical, matrices. External calibration does not inherently compensate for proportional or additive MEs unless they are negligible, whereas MMC can reduce both when matrix matching is sufficiently close. The above calibration strategies mitigate Mes; however, they do not correct temporal signal fluctuations, such as instrumental drift. They are, therefore, often combined with an internal standard (IS), which can compensate for instrumental drift and common matrix-dependent signal changes when the analyte and IS respond similarly [13,16].

Therefore, the first objective of this study was to compare the performance of five calibration strategies (EC, EC-IS, SA, SA-IS and SLSA-IS) for the simultaneous quantification of multiple trace elements, including As, Cd, Cr, Ni, Pb and Se when applied on a mussel tissue CRM. The second objective was to evaluate how the selected calibration strategy performed when used to quantify such trace elements in two crab tissues, including the hepatopancreas and claw meat, which largely vary in terms of matrix and element compositions. The last objective was to explore cross-matrix calibration using both the crab tissue-based and CRM-based MMC for the determination of selected elements in brown crab tissues.

## 2. Materials and Methods

### 2.1. Reference Material and Samples

A mussel tissue CRM, European Reference Material ERM^®^-CE278k (European Commission’s Joint Research Centre, Geel, Belgium), was used in this study. Because no crab tissue CRM certified for the target elements was available, ERM^®^-CE278k was selected as a surrogate seafood-based biological matrix to assess calibration trueness and matrix-related effects. The certified mass fractions ± uncertainty of the six target elements were As 6.7 ± 0.4 mg/kg, Cd 0.336 ± 0.025 mg/kg, Cr 0.73 ± 0.22 mg/kg, Ni 0.69 ± 0.15 mg/kg, Pb 2.18 ± 0.18 mg/kg and Se 1.62 ± 0.12 mg/kg.

Two female brown crabs (*Cancer pagurus*) bought from a local fishmonger in Dublin, Ireland were slaughtered in the store and refrigerated at 4 °C. To facilitate the meat extraction, crabs were treated by high-pressure processing (HPP 300 MPa for 4 min), which was carried out in an industrial HPP unit (vessel volume 420 L, model Hiperbaric 420, Hiperbaric, Burgos, Spain) located at an HPP toll processing facility (HPP Tolling Ltd., St Margarets, Co. Dublin, Ireland) [17,18]. Subsequently, crab shells were removed manually. For each crab, hepatopancreas and claw meat were separated, vacuum packaged and stored at −20 °C before use. Samples were freeze-dried for 72 h (Lyovapor™ L-200, Mason Technology, Co. Dublin, Ireland). Sample weights before and after freeze drying were recorded to calculate water content. Freeze dried samples were crushed, homogenised and stored in a desiccator with silica gel beads.

### 2.2. Sample Digestion and Spiking with Standards

About 0.2 g of CRM or freeze-dried crab sample (exact weight recorded) was weighed into each Teflon vessel along with 8 mL HNO_3_ (67–69%, ROMIL-SpA™, ROMIL Ltd., Cambridge, UK) and 2 mL H_2_O_2_ (35%, Suprapur^®^, Merck KGaA, Darmstadt, Germany). Vessels were then microwave-digested (MARS 6, CEM Corporation, Matthews, NC, USA) through a two-step temperature program [19]. Briefly, the temperature was raised from room temperature to 165 °C within 15 min and held for 2 min, then ramped to 180 °C within 5 min and held for a further 15 min. A total of 18 CRM digests were prepared in three different batches (6 per batch). For brown crab, a total of 24 digests (2 crabs × 2 tissues × 6 spike levels) were prepared in two batches, and each digestion batch included one CRM digest prepared in parallel for quality control.

Digests were transferred to 50 mL centrifuge tubes. For each CRM digestion batch, the 6 digests were assigned to 6 spike levels (0, 1, 5, 10, 20 and 50 µg/L) by spiking with a multi-element standard solution (1 mg/L) made from a 9-element stock solution (ICP multi-element standard solution IX (Certipur^®^, Supelco, Merck KGaA, Darmstadt, Germany; As, Be, Cd, Cr, Hg, Ni, Pb, Se and Tl, each at 100 mg/L). All solutions were internally standardised by adding 100 μL of an indium (In) solution (1 mg/L) made from the stock solution of 1000 mg/L (Supelco, Merck KGaA, Darmstadt, Germany), followed by diluting with 0.5 mol/L (M) HNO_3_ to 50 mL. The same spiking procedure was applied to brown crab tissues to generate tissue-specific SA and SA-IS calibrations.

Aqueous multi-element standards for EC and EC-IS were prepared by spiking with multi-element solutions (1 mg/L) of 0, 0.05, 0.25, 0.5, 2.5 and 5 mL into 50 mL volumetric flasks. Each flask was then internally standardised by adding 100 μL of an In solution (1 mg/L) and diluting with 0.5 M HNO_3_ to obtain final concentrations of 0, 1, 5, 10, 50 and 100 µg/L. The 0 µg/L solution served as the reagent blank.

### 2.3. ICP-MS Analysis

Elemental analysis of all CRM and crab tissue samples was conducted on a Thermo Scientific iCAP Q ICP-MS (Thermo Fisher Scientific, Bremen, Germany). Analyses were performed in five different batches, and each included a set of aqueous-based multi-element standards for EC/EC-IS calibration and a reagent blank. The ICP-MS operation conditions are listed in Table 1.

### 2.4. Calibration Methods

#### 2.4.1. External Calibration Method Without and with Internal Standard (EC and EC-IS)

External Calibration and EC-IS calibration curves were generated separately for each element (As, Cd, Cr, Ni, Pb and Se) using the signal intensities obtained from the six multi-element calibration solutions (0, 1, 5, 10, 50, and 100 µg/L). For EC, the analyte intensity was plotted against the analyte concentration in the aqueous standard solution, with the analyte intensity in the reagent blank (concentration 0 µg/L) as the y-axis intercept of the regression line. For EC-IS, the ratio of the analyte intensity and the IS (In) intensity was plotted against the analyte concentration in the aqueous standard solution, with the intensity ratio in the reagent blank as the y-axis intercept of the regression line. The calibration range was from 0 to 100 µg/L. The element concentration in the sample was calculated using equations obtained from the corresponding calibration curves.

#### 2.4.2. Standard Addition Calibration Method Without and with Internal Standard (SA and SA-IS)

Standard addition levels in CRM/crab tissue digests were expressed on the dry mass basis by normalising to the measured mass of each tissue aliquot. The effective addition level, *C*_add_ (mg/kg, dry weight), was calculated using Equation (1):

(1) $$C_{\mathrm{add}}=\frac{C_{\mathrm{std}}\times V}{m_{\mathrm{dry}}},$$

where *C*_std_ is the SA concentration in the final ICP-MS test dilution (µg/L); *V* is the final dilution volume (L), and *m*_dry_ is the dry sample mass (g).

For SA calibration, the analyte intensity was plotted against *C*_add_, with the analyte intensity in the un-spiked sample as the y-axis intercept (addition level of 0 mg/kg, dry weight) of the regression line. The negative x-intercept (y = 0) of the calibration line corresponded to the endogenous concentration of the analyte in the sample [16]. For SA-IS, the ratio of the analyte intensity and the IS (In) intensity was plotted against the *C*_add_, with the intensity ratio in the un-spiked sample as the y-axis intercept of the regression line. For both SA and SA-IS, the endogenous analyte concentration of the sample matrix used to construct the calibration curve was obtained directly as the absolute value of the negative x-intercept (y = 0) of the SA/SA-IS calibration curve.

The SA-IS calibration curves were also evaluated for cross-matrix application as MMC, whereby a calibration curve generated from one biological matrix was applied to quantify analytes in a different matrix. For a test sample, the concentration difference relative to the calibration matrix was calculated from the corresponding calibration equation, and the total analyte concentration was obtained by adding this difference to the endogenous analyte concentration of the matrix used to construct the calibration curve.

#### 2.4.3. Single Level Standard Addition with Internal Standard (SLSA-IS)

According to Zhu et al. [14], the SLSA-IS approach requires only the data from a non-spiked sample and a single spiked sample for calculation. In the present study, a volumetric method was used for spiking, and the equation used for the calculation of sample concentration was adopted from Zhu et al. [14] and accordingly modified as follows (the deduction has been detailed in Appendix A).

(2) $$C_{x(\mathrm{dry})}=\frac{5\times 10^{-2}\times C_{\mathrm{std}}}{m\times \frac{R_{S}}{R}-m_{S}},$$

where *C*_x(dry)_ is the concentration of the element of interest in the dry sample (mg/kg); *C*_std_ is the SA concentration in the final ICP-MS test dilution (µg/L); *m* is the dry sample mass used to prepare the non-spiked sample dilution (g); *m*_S_ is the mass of dry sample making the spiked sample dilution (g); *R*_S_ is the signal intensity ratio of the element of interest to the IS in the spiked sample; *R* is the signal intensity ratio of the element of interest to the IS in the non-spiked sample.

#### 2.4.4. Calibration Parameters

The background equivalent concentration (BEC) expresses the instrument’s background signal as the equivalent concentration of the analyte in the sample digest dilution (µg/L), serving as a diagnostic metric for system cleanliness and interference. The LoD is the lowest analyte concentration in the final digest dilution that can be confidently distinguished from the blank. The LoQ is the lowest concentration that can be quantified with acceptable precision and trueness. BEC, LoD and LoQ were calculated using Equations (3), (4) and (5), respectively:

(3) $$\mathrm{BEC}=\frac{{\bar{y}}_{\mathrm{blank}}}{S},$$

(4) $$\mathrm{LoD}=\frac{3\times {\mathrm{SD}}_{\mathrm{blank}}}{S},$$

(5) $$\mathrm{LoQ}=\frac{10\times {\mathrm{SD}}_{\mathrm{blank}}}{S},$$

where *S* is the slope of the calibration curve; ${\bar{y}}_{\mathrm{blank}}$ and SD_blank_ are the mean and standard deviation (SD), respectively, of blank signals (cps), as calculated by the Qtegra software (Thermo Fisher Scientific, Bremen, Germany).

The BEC, LoD and LoQ of elements were reported in Table 2 based on three independent EC batches.

### 2.5. Assessment of Matrix Effects (MEs)

Matrix effects were assessed by comparing the slope of the SA calibration curve with that of the EC curve, according to Equation (6), adapted from previous studies [20,21,22].

(6) $$\mathrm{ME}\left(\%\right)=\left(\frac{S_{\mathrm{SA}}}{S_{\mathrm{EC}}}-1\right)\times 100,$$

where *S*_SA_ is the slope of the SA calibration curve (without normalisation of addition to the dry weight but in the ICP test solution); *S*_EC_ is the slope of the aqueous EC standard curve.

Negative and positive ME (%) values indicate signal suppression and signal enhancement extent, respectively.

### 2.6. Accuracy

Accuracy was assessed in terms of trueness and precision, as indicated by the International Organization for Standardization [23]. Trueness was expressed as bias calculated using Equation (7). The intermediate precision was expressed as the relative standard deviation of element concentration replicates (RSD) according to Equation (8). Replicates were obtained within the same laboratory across different days and digestion batches.

(7) $$\mathrm{Bias}(\%)=\left(\frac{\mathrm{measured concentration value in dry base}}{\mathrm{certified concentration value in dry base}}-1\right)\times 100,$$

(8) $$\mathrm{RSD}\left(\%\right)=\frac{\mathrm{SD}}{\mathrm{Mean}}\times 100,$$

where the measured and the certified values in dry base are in mg/kg; SD is the standard deviation of replicate results, and Mean is the corresponding average value.

### 2.7. Statistical Analysis

Calibration curves and calculations were completed in Excel (version 2507). Data visualisation and significance analyses were conducted in R (version 4.3.3). Mean ME values and mean measured elemental concentrations were reported along with 95% confidence intervals (CIs), *n* = 3. For the crab tissue concentration estimates, regression-based 95% CIs were calculated by propagating the 95% confidence limits of the corresponding calibration slope to the original concentration estimate. One-sample *t* test against 0 was used to determine whether the ME (%) calculated for each calibration strategy was significant at a 0.05 significance level. The mean of measured concentrations of each element was compared against the corresponding certified reference value using a two-tailed one-sample *t*-test, with the certified value used as the test value. Statistical significance was determined at *p* < 0.05, 0.01 and 0.001. Within each element, one-way ANOVA was performed, with omnibus *p*-values adjusted across elements using the Holm procedure. Tukey’s HSD test was then applied to elements with significant Holm-adjusted ANOVA results (*p* < 0.05). Results were displayed as significance letters in Supplementary Figure S1.

## 3. Results and Discussion

### 3.1. MEs in the Certified Reference Material (CRM)

**Figure 1:**
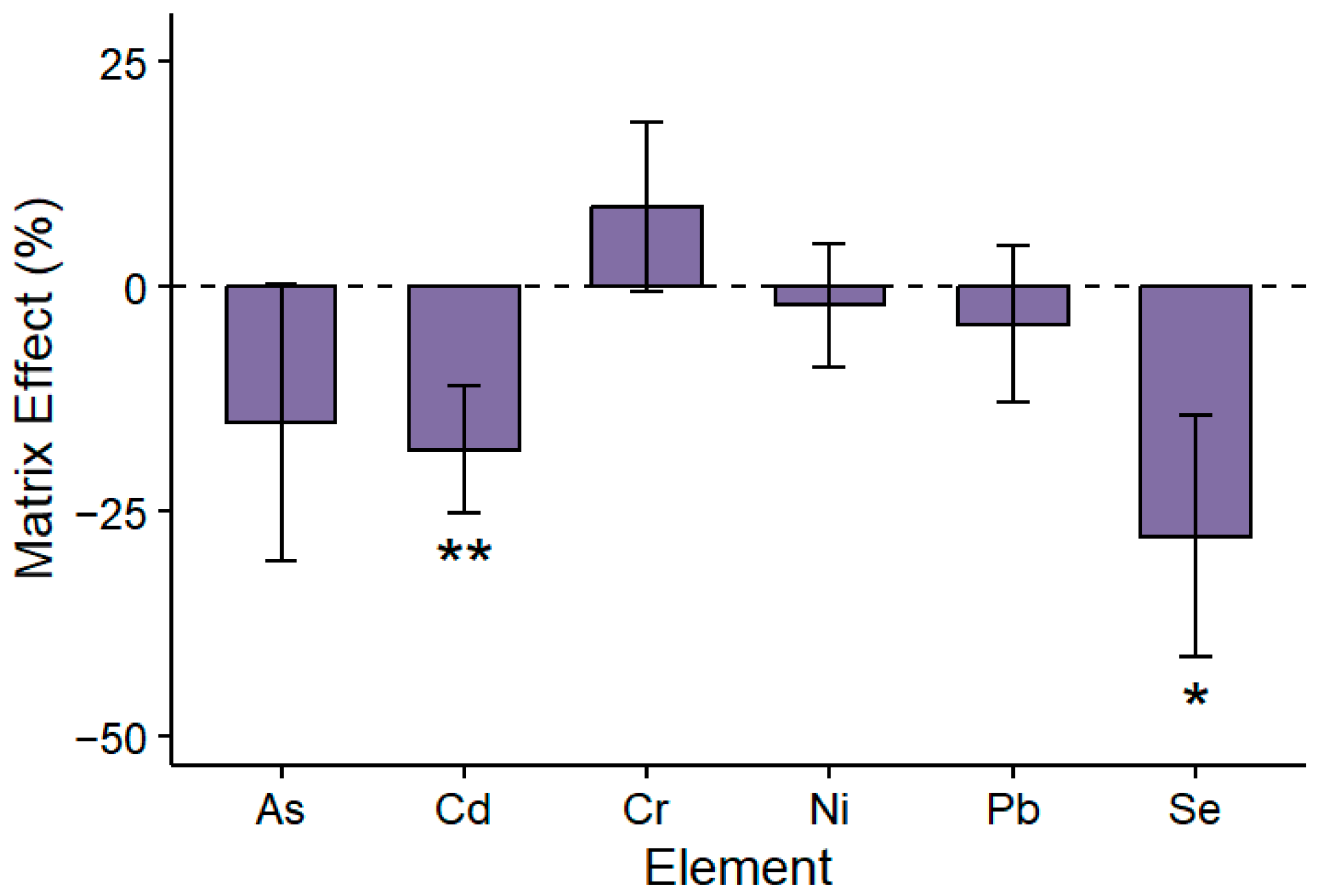
Matrix effect (%) of each element calculated from the slope ratio between standard addition (SA) and external calibration (EC) curves. Bars represent mean matrix effects (*n* = 3), and error bars indicate 95% confidence intervals of the mean. Asterisks indicate statistical significance (*, *p* < 0.05; **, *p* < 0.01) from one-sample *t*-test against 0.

As shown in Figure 1, MEs in the mussel tissue CRM digest were element-dependent. Significant mean signal suppressions were observed for Se (−27.74%; 95% CI: −41.20 to −14.28%) and Cd (−18.09%; 95% CI: −25.17 to −11.01%). While a signal suppression of −15.12% was obtained for As, its 95% CI was wider and included zero (−30.57 to 0.33%, *p* = 0.052), indicating greater uncertainty of this estimate. In contrast, Cr, Ni and Pb were only weakly affected by the CRM matrix (*p* > 0.05). Exact effect estimates, 95% CIs and *p*-values are provided in Supplementary Table S1. Such element-specific MEs have been widely reported in ICP-MS [10,15,16,24,25,26].

Matrix effects may arise from changes in aerosol formation and transport, plasma conditions and space-charge-related ion losses, leading to altered sensitivity in digests relative to aqueous standards [10,15,26]. In the current study, microwave digests, even after dilution, contained additional matrix components relative to the aqueous standards, including residual carbon, dissolved salts and other remaining tissue-derived components. Carbon-containing species in the plasma have been reported to alter the ionisation efficiency and signal responses of elements with high first ionisation potentials (FIPs), such as As, Se, Te and Hg [13,27,28]. In addition to these non-spectral effects, matrix-induced spectral interferences may also contribute to the observed signal changes. For instance, ^52^Cr, ^75^As and ^78^Se are well recognised as being particularly susceptible to polyatomic interferences, such as ^40^Ar^12^C+, ^36^Ar^16^O+, ^40^Ar^35^Cl+ and ^40^Ar^38^Ar+ in biological and chloride-containing matrices [13]. In the current study, these isotopes were measured in kinetic energy discrimination mode to reduce such polyatomic interferences. Nevertheless, residual spectral contributions may not be completely eliminated [29]. Notably, Cd, a low FIP element, still showed significant suppression, while a slight enhancement was observed for Cr. Therefore, the observed matrix-dependent signal changes might not be governed by a single matrix component but rather reflect the combined influence of both non-spectral MEs and residual spectral interferences [10,13,15,26].

These findings indicated that EC was inadequate for accurate multi-element determination in seafood matrix, particularly for As, Cd and Se. Therefore, alternative calibration strategies were compared to identify a reliable solution.

### 3.2. Analytical Performance of Calibration Strategies for Multi-Element Determination in the CRM

**Figure 2:**
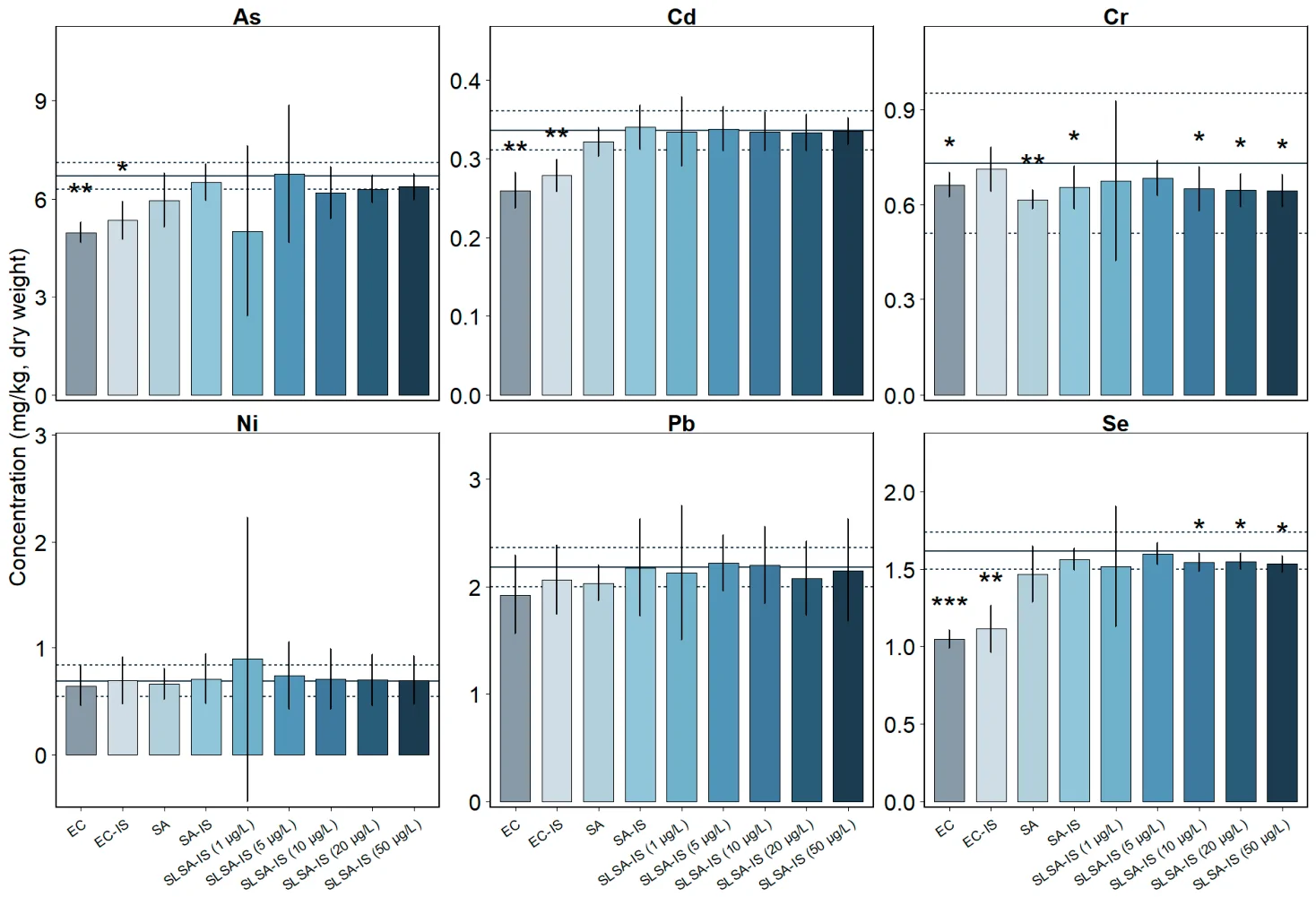
Measured elemental concentrations under different calibration strategies. Bars display the mean concentration (dry weight, mg/kg), *n* = 3. Error bars represent 95% confidence intervals of the mean. For each element, the horizontal solid line indicates the CRM certified value and dashed lines denote the supplier-reported uncertainty range. Asterisks mark the significant difference between the measured and certified values (*, *p* < 0.05; **, *p* < 0.01; *** *p* < 0.001). Bar colours represent analytical methods. EC, external calibration; EC-IS, external calibration with internal standard; SA, standard addition; SA-IS, standard addition with internal standard; SLSA-IS (1–50 µg/L), single-level standard addition with internal standard at the indicated spike levels.

Five calibration strategies (EC, EC-IS, SA, SA-IS and SLSA-IS) were evaluated in the CRM mussel tissue by using precision (RSD), as shown in Table 3, and trueness (relative bias to the certified value) in Table 4. The measured concentrations were compared with the certified values and ranges in Figure 2. Trueness was primarily evaluated against the AOAC bias-acceptance criterion, while the one-sample *t*-test (Figure 2) was used as complementary statistical evidence of deviation from the certified value.

#### 3.2.1. External Calibrations (EC and EC-IS)

For all elements in Table 3, EC generally produced acceptable repeatability with RSDs (2.28 to 11.78%) less than 15% [30]. The acceptability of trueness varied depending on elements. As shown in Figure 2, the average measured concentrations with the 95% CIs of As, Cd and Se were considerably below the lower limits of the certified ranges, with negative biases exceeding 20% (Table 4). These measurement values were unacceptable according to AOAC guidelines [30], which establish that bias values should be between −20% and 10% for analyte concentrations ≥ 0.1 mg/kg [30]. For Pb, although a relatively small bias was observed, the measurement was still outside the certified range (Figure 2). In contrast, the measured Cr and Ni in Figure 2 fell within the certified ranges with the acceptable AOAC bias range of −20% to 10%.

The addition of In as the IS achieved acceptable RSDs for all elements (2.99–12.82%) (Table 3) and slightly reduced biases relative to EC (Table 4). For Cr, Ni and Pb, EC-IS determined concentrations within the certified ranges and closer to the certified values than EC (Figure 2). However, for elements that show matrix-induced signal changes, including As, Cd and Se, although limited biases were observed by EC-IS, the determinations were still substantially lower than the certified ranges, because the primary benefit of IS in the calibration is tracking the short-term instrumental drift and its effect on correction of MEs is usually ineffective or insufficient [15,31].

#### 3.2.2. Standard Addition Calibrations (SA, SA-IS and SLSA-IS)

Compared to aqueous calibrations, calibrations based on standard addition (SA and SA-IS) markedly improved multi-element trueness while maintaining similar precisions (RSDs ≤ 15%), particularly for As, Cd and Se whose signals were significantly affected by MEs (Figure 1). Furthermore, SA measured Cd within the certified range, while the As and Se concentrations were outside of the ranges (Figure 2), but biases were smaller than EC and EC-IS (Table 4), suggesting that SA effectively compensates for MEs by calibrating within the sample matrix [13,16]. Compared with SA, SA-IS further reduced biases (Table 4) and obtained measurements within certified ranges for all elements (Figure 2). Therefore, the combination of matrix compensation with IS achieved the best analytical performance.

An advantage of SLSA and SLSA-IS is that it does not require the preparation of multiple spiked calibration levels; however, its analytical performance may depend on the selected spike level [14,16]. At the lowest spiked concentration (1 µg/L), the SLSA-IS underestimated the concentration of As (Figure 2), and overall, it showed large variability among the three replicates, resulting in the wide 95% CI in Figure 2 and large RSDs in Table 3, especially for As, Cr and Ni. In contrast, both precision and trueness were improved substantially with SLSA-IS at higher spikes (Table 3 and Table 4). Furthermore, SLSA-IS at 5–50 µg/L produced concentrations close to those determined by SA-IS for all elements, with no significant differences between the measurement means provided by these two calibration strategies (*p* ≥ 0.05, Supplementary Figure S1). Although SLSA-IS (5–50 µg/L) provided acceptable biases of −11.72–7.21% for all elements (Table 4), RSDs at the spike of 5 µg/L and 10 µg/L for Ni were large (17.27% and 15.97% in Table 3), and the measured As at the spike of 10 µg/L did not fall within the certified range (Figure 2). In general, the spike of 20–50 µg/L provided more stable performance. This phenomenon may be attributed to the use of very low standard spiking levels. At low spike concentrations, small variations in the added standard can result in relatively large variations in the expected analyte concentrations, leading to lower method precision [14,32,33].

Overall, among the five methods, SA, SA-IS and SLSA-IS at 20–50 µg/L were the only calibration strategies that had a bias value within the acceptable range of −20–10% and RSD ≤ 15% across all elements. However, SA-IS was the only calibration strategy to provide concentration measurements for each element within the certified range of the reference material. Therefore, the current CRM analysis identified SA-IS as the most promising strategy among the investigated strategies, supporting its subsequent evaluation in brown crab tissues.

### 3.3. Quantification of Multiple Elements in Brown Crab Hepatopancreas and Claw Meat

**Figure 3:**
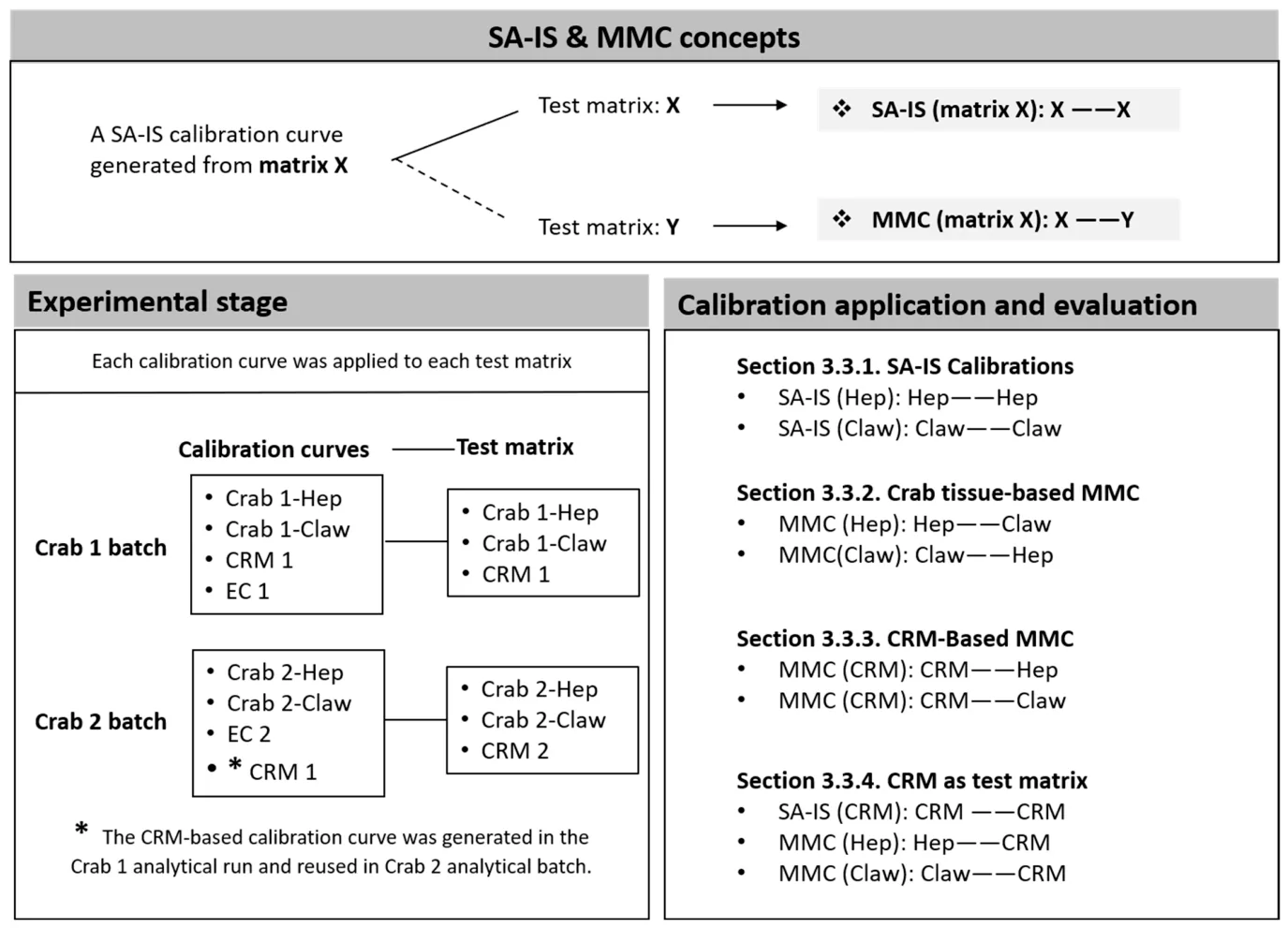
Schematic overview of the standard addition-internal standard (SA-IS) and matrix-matched calibration (MMC) framework. Hep, hepatopancreas of brown crab (*Cancer pagurus*); Claw, claw meat of brown crab; CRM, certified reference material (mussel tissue).

Before discussing the crab results, the terminology and calibration applications used for SA-IS and MMC are clarified in Figure 3. In this study, SA-IS denotes a matrix-specific standard-addition calibration constructed within the same sample matrix being quantified, whereas MMC refers to cross-matrix application of an existing SA-IS calibration curve to a different biological matrix. Direct evidence for cross-matrix transfer of MMC in solution-based ICP-MS remains limited. Therefore, the present cross-matrix comparison was undertaken as an exploratory assessment of calibration transferability across different seafood matrices.

The hepatopancreas is a metabolically active digestive and detoxification gland and is widely recognised as a preferential reservoir for many trace elements, whereas claw is characterised as protein-rich and relatively less prone to the accumulation of mineral elements [34,35]. These two crab tissues thereby offered complementary test matrices of different biochemical and elemental compositions.

#### 3.3.1. SA-IS Calibrations

As shown in Table 5, SA-IS produced good linearity in most cases (*R^2^* ≥ 0.99), except for As. Hepatopancreas- and claw-based SA-IS generated As calibration curves with *R^2^* of 0.8065–0.9902. Although As is inherently challenging for ICP-MS because of its high FIP, susceptibility to spectral interferences and monoisotopic nature [36], it was accurately determined in the mussel CRM. Therefore, the poor As performance in crab tissue was more directly attributed to the mismatch between the high native analyte concentration and the calibration range. In SA-IS, the native elemental concentration was obtained from the x-intercept (y = 0) of a linear regression. Therefore, the accuracy was critically dependent on the spiking concentrations [16,37,38]. In this study, the native As concentrations in the final crab tissue digest dilutions were high (150–300 µg/L) and exceeded the spike range of 0–50 µg/L. In this case, the signal increment from the spike was negligible relative to the high-intensity native signal, and the calculated regression slope became susceptible to minor instrumental noise and plasma fluctuations, which might explain the erratic results for As in Table 6. In contrast, the mussel CRM’s native As concentration (about 27 µg/L) produced an appropriate spike-to-native ratio within the 0–50 µg/L range (Section 3.2.2). This ensured a significant increase in signal strength in a robust regression model and a brief and reliable extrapolation, allowing for accurate results. Therefore, the present SA range was not adequate for reliable quantitative determination of As in the crab tissues. Accordingly, crab-tissue As concentrations were excluded from quantitative interpretation and from the subsequent MMC comparisons.

Subsequently, elements in hepatopancreas and claw were analysed using SA-IS calibrations (matrix source of hepatopancreas and claw, respectively in Table 6) and compared with the EC determination. As shown in Table 6, for Crab 1 hepatopancreas, Cd and Se concentrations were determined as 9.71 and 6.76 mg/kg (dry weight), respectively, by the SA-IS method, while EC determined 23% and 19% lower concentrations (7.43 and 5.46 mg/kg, dry weight of Cd and Se, respectively). In contrast, EC gave a 20% higher Cr concentration than SA-IS (Hep), while for Ni and Pb, estimations by EC were close to that by SA-IS (Hep). This was similar to the pattern found in the previous CRM analysis in Figure 1, which showed ME-induced signal suppression for Cd and Se and signal enhancement for Cr. Similarly, for Crab 1 claw, SA-IS (Claw) increased the EC determination of Cd from 0.012 to 0.015 mg/kg (dry weight) and Se from 6.46 to 8.58 mg/kg (dry weight), while decreasing the Cr estimation by EC from 0.036 to 0.031 mg/kg (dry weight). Smaller differences of estimations for Ni and Pb were found between SA-IS (Claw) and EC. Consistent results were observed for Crab 2 hepatopancreas and claw.

To facilitate comparison with other published studies, the elemental concentrations of crab tissues determined by SA-IS were reported on both dry-weight (Table 6) and wet-weight bases (Supplementary Table S2). Among elements, As (measured by EC) was present at the highest level in claw meat and hepatopancreas for Crab 1 and Crab 2, showing the strong bioaccumulation tendency of As in crab tissues [5,35,39], followed by Cd and Se. Nickel, Cr and Pb were at much lower levels. To summarise the published data of brown crab from different geographical areas, As, Cd, Cr, Ni, Pb and Se ranged from 16–50, 0.005–0.07, 0.012–0.016, 0.06–0.06, 0.006–0.03, 0.88–1.75 mg/kg (wet weight), respectively for muscle meat and 14–49, 2–30, 0.025–0.2, 0.5–1.5, 0.02–0.1, 1.1–2 mg/kg (wet weight), respectively for brown meat or hepatopancreas [40,41,42,43]. Additionally, Ervik et al. [44] estimated 44.2 (As), 10.3 (Cd), 0.18 (Cr), 0.79 (Ni), 0.16 (Pb) and 4.83 (Se) mg/kg (dry weight) for brown meat. Generally, the elemental concentrations and distribution patterns obtained in this study by SA-IS were broadly comparable with previous reports.

Overall, the application of SA-IS in two representative edible crab tissues (hepatopancreas and claw) indicated that SA-IS was applicable to complex crab tissues for most trace elements, including Cd, Cr, Ni, Pb and Se. However, its applicability to samples containing extremely high native elemental concentrations, such as As, requires careful consideration to ensure that the calibration range adequately encompasses the analyte concentration.

#### 3.3.2. Crab Tissue-Based Matrix-Matched Calibration (MMC)

This section explored cross-tissue calibration transfer by applying MMC (Claw) to hepatopancreas and MMC (Hep) to claw meat (Table 6). The resulting concentrations were compared with the corresponding same-matrix SA-IS estimates.

For the analysis of hepatopancreas samples, MMC (Claw) produced Cr, Ni and Pb determinations comparable to those obtained by SA-IS (Hep). For Cd and Se, MMC (Claw) estimated concentrations that were approximately 7% lower and 3–6% higher, respectively, than those determined by SA-IS (Hep). The differences in Cd, Cr and Se determinations between MMC (Claw) and SA-IS (Hep) were remarkably smaller than those between EC and SA-IS (Hep). In contrast, SA-IS (Hep), MMC (Claw) and EC yielded comparable results for Pb and Ni. Furthermore, similar patterns were observed for elemental determinations of claw samples using MMC (Hep).

Overall, crab tissue-based MMC showed closer agreement with SA-IS than EC. However, the degree of agreement varied among elements and tissues.

#### 3.3.3. CRM-Based MMC

This section explored the possibility of using an MMC curve generated from mussel tissue CRM spiked with different levels of multi-element standard solution for the determination of selected elements in hepatopancreas and claw meat.

As shown in Table 6, for Crab 1 hepatopancreas and claw, MMC (CRM) determined the Cd and Se concentrations 17–20% and 38–40% higher than EC, respectively. For other elements, MMC (CRM) produced concentrations that were 6–9% lower for Cr, 5–9% higher for Pb and 2–10% higher for Ni than those obtained by EC. Compared with SA-IS, MMC (CRM) estimated the largest difference for hepatopancreas Se (11% higher) and claw Cr (10% higher).

For Crab 2, MMC (CRM) also increased Cd and Se estimates relative to EC, by approximately 13–20% and 34–39%, respectively. However, compared with Crab 1, larger deviations from SA-IS were observed for Cr and Pb in Crab 2 claw meat. This might be due to the uncertainty introduced by the cross-batch application of the calibration curve. Overall, MMC (CRM) partly mitigated the EC underestimates of Cd and Se, while batch-specific validation was necessary. Crab tissue-based MMC generally produced results closer to SA-IS than CRM-based MMC.

#### 3.3.4. CRM as Test Sample

Because the elemental concentrations in the CRM are traceable to certified values, the CRM was further used as an independent test sample to evaluate calibration strategies, including SA-IS, crab tissue-based MMC and EC. The assessment was based on the bias between the measured CRM value and the corresponding certified value.

For Crab 1, SA-IS (CRM) produced the closest agreement with the certified values for As, Cd and Se with biases of −2.01%, 2.06% and −2.82%, respectively. In contrast, EC underestimated these elements with biases of −23.57%, −20.07%, and −34.11%, respectively. These results were consistent with the CRM analysis in Section 3.2. When using crab tissue-based MMC for CRM quantification, MMC (Claw) estimated Cd and Se with acceptable biases of 3.57% and −8.07%, respectively. However, MMC (Hep) estimated Cd and Se with relatively larger biases of 11.53% and −12.79%, respectively. The discrepancies observed among calibrations of MMC (Hep), MMC (Claw) and SA-IS (CRM) suggest that, although hepatopancreas, claw meat and mussel CRM are all seafood tissues, their calibration responses after digestion are not fully interchangeable. These comparisons indicated that when the calibration matrix was identical to the test CRM matrix (SA-IS), accuracy was substantially improved, highlighting the importance of matrix compatibility for elements vulnerable to MEs such as Cd and Se.

For Crab 2, the CRM test required separate consideration. In this batch, EC and crab tissue-based MMC were established from Crab 2 ICP-MS analysis, while the SA-IS curve (CRM) used for the determination of Crab 2 hepatopancreas, claw meat and CRM was generated in the Crab 1 analytical run. For Crab 2-CRM, the transferred SA-IS (CRM) still gave the best agreement with the certified As value, with a bias of −4.22%, which was markedly better than crab tissue-based MMC and EC. For Se, although the bias of the transferred SA-IS (CRM) was relatively larger than that observed in the Crab 1 batch, it was still smaller than the biases for EC and MMC based on crab tissue from Crab 2. However, for Cd, the transferred SA-IS (CRM) also improved the estimate relative to EC, although crab tissue-based MMC produced smaller biases. These results indicated that SA-IS (CRM) is advantageous when applied within the same analytical batch, but inter-run calibration transfer might introduce additional uncertainty.

For the analysis of Cr, Ni and Pb of both Crab 1 and Crab 2, EC determined these elements close to the certified values with small biases among calibrations in Table 6, which was consistent with the CRM analysis in Table 4. For Crab 1 analysis, although SA-IS (CRM) obtained relatively larger biases compared with EC, the measured Cr, Ni and Pb values of CRM were all within the certified ranges, while crab tissue-based MMC tested Pb values of 2.38 and 2.39 mg/kg, which were slightly outside the range (2.00–2.36 mg/kg). In contrast, for Crab 2, SA-IS (CRM) determined Pb of 2.00 mg/kg at the bottom of the certified range (2.00 mg/kg), which might be reasonably caused by the calibration error when the SA-IS (CRM) curve was applied across independent ICP-MS runs. The larger CRM measurement biases of SA-IS (CRM) for Crab 2 than Crab 1 were observed for most elements in Table 6. This was similar to the MMC (CRM) analysis of crab tissues from Crab 1 and Crab 2 in Section 3.3.3. Then, the need to reduce batch-to-batch calibration variation was suggested to ensure accurate calibration performance [45,46].

Overall, for the CRM tests of Crab 1 batch, among the tested calibrations, SA-IS (CRM) determined all elements within the certified ranges and measured As, Cd and Se closest to the certified values. Crab tissue-based MMC could approximate the SA-IS (CRM) measurements of Cd and Se, while its determination of As should be re-assessed in the future after improving the calibration curve quality. By contrast, EC showed reliable determination of Cr, Ni and Pb but remarkable underestimations of As, Cd and Se. The Crab 2 analysis of CRM indicated the importance of batch-specific validation of calibration.

The application of SA-IS in homogeneous and identical matrices has already been proposed by Carter et al. [16] with the experimental support of a study by Christopher et al. [47]. Christopher et al. [47] showed the feasibility of using SA-IS for the determination of multiple elements in a fish tissue CRM and in whole-blood CRMs. Despite the advances of SA being well recognised by previous publications, its drawback of low throughput due to the requirement for building a calibration curve for each sample limited its application to the analysis of a large number of samples [16]. The present results suggest that cross-matrix MMC may be feasible for some selected matrix-element applications. However, further work should provide more experimental repetitions and widen the tissue range and crab species (even other crustaceans) to confirm the robustness and transferability of this calibration strategy.

### 3.4. Study Limitations

Several limitations should be considered when interpreting the present findings. First, CRM comparison was based on three independent calibration batches, while the real-crab tissue evaluation included two individual crabs. Although 95% CI and regression-based uncertainty were reported, measurement repeatability, intermediate precision and generalisability across natural biological variability remain to be established. In addition, the measurement uncertainty to take into account the precision, bias and other relevant sources of uncertainty was also beyond the present experimental design.

Given the limited direct precedent for cross-tissue calibration transfer and the absence of crab-tissue CRM, the crab-tissue SA-IS and MMC results could not be independently verified against an external benchmark. In this case, the present real-tissue evaluation (*n* = 2, without independent replication) should therefore be regarded as exploratory. Moreover, the CRM-derived calibration was reused across analytical batches for Crab 2, indicating that batch-specific validation is required before routine use.

Finally, some element-specific analytical constraints remain. Relevant results should therefore be cautiously interpreted. Native As concentrations exceeded the applied SA range, increasing extrapolation uncertainty. An extended addition range or additional sample dilution would be required for reliable crab-tissue As determination. Moreover, the use of a single IS (In) may not equally compensate for ME across all analytes, while multiple internal standards spanning the analyte mass range can provide more element-specific correction in multi-element ICP-MS analysis. As all crab tissues were obtained following HPP treatment and no non-HPP-treated control was included in the present study, the potential influence of HPP-pretreatment on matrix-dependent calibration behaviour could not be assessed against conventionally handled tissues.

## 4. Conclusions

This study compared five calibration strategies (EC, EC-IS, SA, SA-IS, SLSA-IS) for ICP-MS quantification of selected trace elements, including As, Cd, Cr, Ni, Pb and Se, using a CRM mussel tissue and subsequently brown crab hepatopancreas and claw muscle tissues, representing brown and white meat, respectively. It was found that MEs caused a systematic underestimation of Se, Cd and As when aqueous-based calibrations (EC) were used. SA compensated for these MEs to a great extent. The combination of IS further improved trueness. Among all calibrations, SA-IS provided the best overall performance. When applied to hepatopancreas and claw, SA-IS performed satisfactorily for the determination of Cd, Cr, Ni, Pb and Se, while As could not be reliably quantified under the present calibration design.

The feasibility of using crab tissue-based MMC and CRM-based MMC was also explored. For Cd and Se, SA-IS provided the closest result to the certified concentration, followed by MMC, while EC showed the largest deviations. For crab tissue analysis, the crab tissue-based MMC produced results broadly comparable to SA-IS, while CRM-based MMC showed larger deviations for Cd and Se. In contrast, Cr, Ni and Pb were less affected by the calibration matrix, with similar estimates obtained across the different calibration approaches.

Overall, among the calibration strategies tested in this study, SA-IS showed the best overall performance for multi-element determination in brown crab tissues. These findings provide practical insights for future research. The cross-matrix MMC evaluation provided preliminary evidence of calibration transfer for selected matrix-element combinations. However, the observed deviations indicate that such transfer requires matrix- and batch-specific verification before routine application.

## Figures and Tables

**Table 1:** ICP-MS operation parameters and measurement conditions.

Parameter	Setting
Spray chamber	Cyclonic
Nebuliser	Microflow PFA ST (Elemental Scientific Inc., Omaha, NE, USA)
Interface	Ni cones
Mass analyser	Quadrupole
RF Power (W)	1550
Argon gas flow rates (L/min):	
Cool	14
Auxiliary	0.8
Sample	1.13
Spray chamber temperature (°C)	2.5
Maximum oxide and double charge production	^140^Ce^16^O^+^/^140^Ce < 2.2%, ^137^Ba^2+^/^137^Ba^+^ < 3.0%
Replicate samples per isotope per sample	200 (20 runs × 10 sweeps)
Isotope-specific acquisition conditions	^52^Cr, ^60^Ni, ^75^As, ^78^Se, ^111^Cd, ^208^Pb and ^115^In (internal standard)
^52^Cr	Dwell time: 20 ms; KED mode; He: 4.8 mL/min
^60^Ni	Dwell time: 20 ms; KED mode; He: 4.8 mL/min
^75^As	Dwell time: 200 ms; KED mode; He: 4.8 mL/min
^78^Se	Dwell time: 200 ms; KED mode; He: 4.8 mL/min
^111^Cd	Dwell time: 20 ms; KED mode; He: 4.8 mL/min
^208^Pb	Dwell time: 20 ms; STD mode; no collision gas
^115^In (internal standard)	Dwell time: 10 ms; STD mode; no collision gas

KED mode, kinetic energy discrimination mode; STD mode, standard mode.

**Table 2:** Summary of external calibration (EC) parameters (mean ± SD, *n* = 3).

Element	Slope	*R^2^*	BEC (µg/L)	LoD (µg/L)	LoQ (µg/L)
As	1997.1385 ± 197.1889	0.9998 ± 0.0003	0.0077 ± 0.0043	0.0040 ± 0.0019	0.0133 ± 0.0065
Cd	9806.9186 ± 423.3969	0.9999 ± 0.0001	0.0007 ± 0.0003	0.0017 ± 0.0004	0.0058 ± 0.0014
Cr	18,889.6787 ± 1950.5842	0.9999 ± 0.0002	0.0204 ± 0.0097	0.0062 ± 0.0017	0.0207 ± 0.0057
Ni	6905.6516 ± 594.7063	1.0000 ± 0.0001	0.1475 ± 0.1195	0.0276 ± 0.0179	0.0921 ± 0.0598
Pb	134,090.4412 ± 51,028.6709	0.9999 ± 0.0001	0.0069 ± 0.0025	0.0016 ± 0.0005	0.0053 ± 0.0016
Se	281.0526 ± 31.7944	0.9999 ± 0.0002	0.0440 ± 0.0041	0.0292 ± 0.0039	0.0972 ± 0.0131

SD, standard deviation across three independent batches; *R^2^*, coefficient of determination; BEC, background equivalent concentration; LoD, limit of detection; LoQ, limit of quantification. The reported SD is distinct from SD_blank_ used in the LoD and LoQ calculations.

**Table 3:** Relative standard deviation (RSD, %) of the concentration measurement for trace elements in a certified reference material (mussel tissue ERM^®^-CE278k, *n* = 3) under different calibration methods.

Element	RSD (%)								
	EC	EC-IS	SA	SA-IS	SLSA-IS				
					(1 µg/L)	(5 µg/L)	(10 µg/L)	(20 µg/L)	(50 µg/L)
As	2.44	4.45	5.55	3.47	20.93	12.47	5.17	2.65	2.48
Cd	3.48	2.99	2.26	3.34	5.31	3.30	2.93	2.12	2.60
Cr	2.29	3.90	1.96	4.15	14.95	3.21	4.28	3.24	3.19
Ni	11.78	12.82	8.74	13.06	59.89	17.27	15.97	13.96	13.02
Pb	7.72	6.39	3.38	8.46	11.87	4.69	6.58	6.76	8.95
Se	2.28	5.51	5.03	1.79	10.34	1.70	1.54	1.34	1.32

EC, external calibration; EC-IS, external calibration with internal standard; SA, standard addition; SA-IS, standard addition with internal standard; SLSA-IS, single-level standard addition with internal standard (spike level in the bracket).

**Table 4:** Bias (%) of the measured concentration value relative to the reported value in a certified reference material (mussel tissue ERM^®^-CE278k) under different calibration methods.

Element	Bias (%)								
	EC	EC-IS	SA	SA-IS	SLSA-IS				
					(1 µg/L)	(5 µg/L)	(10 µg/L)	(20 µg/L)	(50 µg/L)
As	−25.66	−20.16	−11.09	−2.91	−25.19	1.08	−7.69	−5.88	−4.82
Cd	−22.63	−17.03	−4.34	1.20	−0.49	0.59	−0.51	−0.26	−0.09
Cr	−9.17	−2.47	−15.48	−10.34	−7.54	−6.29	−10.95	−11.46	−11.72
Ni	−6.77	0.66	−3.78	2.92	29.91	7.21	2.85	1.10	0.84
Pb	−11.82	−5.47	−6.91	−0.30	−2.44	1.74	0.79	−4.86	−1.49
Se	−35.54	−31.47	−9.45	−3.42	−6.36	−1.11	−4.57	−4.24	−5.27

Negative values indicate underestimation, while positive values represent overestimation. EC, external calibration; EC-IS, external calibration with internal standard; SA, standard addition; SA-IS, standard addition with internal standard; SLSA-IS, single-level standard addition with internal standard (spike level in the bracket).

**Table 5:** Calibration parameters of brown crab (*Cancer pagurus*) hepatopancreas (Hep)-, claw meat (Claw)- and CRM-based standard addition with internal standard (SA-IS) and external calibration (EC).

Element	Method	Crab No.	R^2^	Calibration Curve Slope
As	SA-IS (Hep)	1	0.8202	0.0510
		2	0.9902	0.0390
	SA-IS (Claw)	1	0.8065	0.0315
		2	0.9479	0.0373
	SA-IS (CRM)	1	0.9996	0.0285
		2		
	EC	1	>0.9999	2117.8371
		2	>0.9999	2018.9630
Cd	SA-IS (Hep)	1	0.9984	0.1261
		2	0.9995	0.1186
	SA-IS (Claw)	1	0.9998	0.1358
		2	0.9997	0.1271
	SA-IS (CRM)	1	>0.9999	0.1378
		2		
	EC	1	>0.9999	10,198.1205
		2	>0.9999	8832.7625
Cr	SA-IS (Hep)	1	0.9995	0.3830
		2	>0.9999	0.3704
	SA-IS (Claw)	1	0.9986	0.3815
		2	0.9996	0.3771
	SA-IS (CRM)	1	0.9999	0.3507
		2		
	EC	1	>0.9999	19,655.7053
		2	>0.9999	19,393.0408
Ni	SA-IS (Hep)	1	0.9995	0.1212
		2	0.9964	0.1180
	SA-IS (Claw)	1	0.9991	0.1195
		2	0.9997	0.1194
	SA-IS (CRM)	1	>0.9999	0.1135
		2		
	EC	1	>0.9999	7150.4402
		2	>0.9999	7012.1562
Pb	SA-IS (Hep)	1	>0.9999	1.1982
		2	0.9999	1.1463
	SA-IS (Claw)	1	0.9998	1.1914
		2	0.9978	1.1493
	SA-IS (CRM)	1	0.9991	1.2284
		2		
	EC	1	>0.9999	79,467.0989
		2	0.9999	65,349.3401
Se	SA-IS (Hep)	1	0.9976	0.0039
		2	0.9994	0.0037
	SA-IS (Claw)	1	0.9910	0.0037
		2	0.9985	0.0036
	SA-IS (CRM)	1	>0.9999	0.0035
		2		
	EC	1	>0.9999	298.7297
		2	>0.9999	266.8788

The CRM-based calibration curve generated in the Crab 1 analytical run was applied to Crab 2. EC slopes were based on analyte intensity versus aqueous standard concentration, whereas SA-IS slopes were based on analyte/indium intensity ratio versus standard-addition level.

**Table 6:** Concentrations (mg/kg, dry weight) of trace elements in brown crab (*Cancer pagurus*) hepatopancreas (Hep), claw meat (Claw) and certified reference material (CRM, mussel tissue ERM^®^-CE278k) quantified using standard addition with internal standard (SA-IS), matrix-matched calibration (MMC), and external calibration (EC).

Element	Test Tissue	Crab 1				Crab 2			
		Matrix Source of Calibration Curve				Matrix Source of Calibration Curve			
		Hep	Claw	CRM	EC	Hep	Claw	CRM	EC
As	Hep	30.29 (18.30–87.90)	49.04 (33.35–92.60)	54.20 (53.08–55.37)	45.14 (44.86–45.43)	29.03 (25.40–33.87)	30.35 (21.77–50.10)	39.72 (38.90–40.58)	31.92 (31.84–32.00)
	Claw	55.45 (33.50–160.91)	89.78 (61.06–169.52)	99.23 (97.18–101.37)	81.23 (80.72–81.75)	36.52 (31.96–42.60)	38.19 (27.39–63.05)	49.98 (48.95–51.06)	38.74 (38.64–38.84)
	CRM (6.7 ± 0.4)	3.67 (2.22–10.65)	5.94 (4.04–11.22)	6.57 (6.43–6.71)	5.12 (5.09–5.15)	4.69 (4.10–5.47)	4.90 (3.51–8.09)	6.42 (6.29–6.56)	5.13 (5.11–5.14)
Cd	Hep	9.71 (8.32–11.66)	9.02 (8.90–9.14)	8.89 (8.87–8.91)	7.43 (7.40–7.47)	6.36 (6.13–6.61)	5.93 (5.83–6.03)	5.47 (5.46–5.49)	4.86 (4.84–4.88)
	Claw	0.016 (0.014–0.019)	0.015 (0.015–0.015)	0.014 (0.014–0.014)	0.012 (0.012–0.012)	0.063 (0.061–0.065)	0.058 (0.057–0.059)	0.055 (0.055–0.055)	0.046 (0.046–0.046)
	CRM (0.336 ± 0.025)	0.37 (0.32–0.44)	0.35 (0.35–0.35)	0.34 (0.34–0.34)	0.27 (0.27–0.27)	0.34 (0.33–0.35)	0.32 (0.31–0.33)	0.29 (0.29–0.29)	0.26 (0.26–0.26)
Cr	Hep	0.091 (0.088–0.094)	0.091 (0.088–0.095)	0.099 (0.097–0.101)	0.109 (0.108–0.110)	0.074 (0.073–0.075)	0.073 (0.071–0.075)	0.088 (0.087–0.090)	0.082 (0.081–0.082)
	Claw	0.031 (0.030–0.032)	0.031 (0.030–0.032)	0.034 (0.033–0.035)	0.036 (0.036–0.036)	0.042 (0.042–0.042)	0.041 (0.040–0.042)	0.054 (0.053–0.055)	0.044 (0.044–0.045)
	CRM (0.73 ± 0.220)	0.60 (0.58–0.62)	0.61 (0.59–0.63)	0.66 (0.65–0.67)	0.68 (0.67–0.69)	0.65 (0.64–0.66)	0.64 (0.63–0.65)	0.70 (0.69–0.71)	0.70 (0.70–0.71)
Ni	Hep	0.47 (0.45–0.49)	0.47 (0.46–0.48)	0.50 (0.49–0.51)	0.49 (0.48–0.49)	0.50 (0.47–0.53)	0.49 (0.48–0.50)	0.52 (0.51–0.53)	0.48 (0.48–0.48)
	Claw	0.049 (0.047–0.051)	0.050 (0.049–0.052)	0.053 (0.052–0.054)	0.048 (0.047–0.048)	0.058 (0.055–0.062)	0.058 (0.057–0.059)	0.058 (0.057–0.059)	0.055 (0.054–0.055)
	CRM (0.69 ± 0.15)	0.74 (0.71–0.77)	0.75 (0.73–0.77)	0.79 (0.77–0.81)	0.72 (0.72–0.73)	0.76 (0.71–0.81)	0.75 (0.74–0.76)	0.78 (0.76–0.80)	0.72 (0.72–0.73)
Pb	Hep	0.12 (0.12–0.12)	0.12 (0.12–0.12)	0.12 (0.12–0.12)	0.11 (0.11–0.11)	0.26 (0.26–0.26)	0.26 (0.25–0.27)	0.28 (0.27–0.29)	0.26 (0.26–0.26)
	Claw	0.040 (0.040–0.040)	0.040 (0.039–0.041)	0.039 (0.038–0.040)	0.036 (0.036–0.037)	0.049 (0.048–0.050)	0.048 (0.046–0.051)	0.081 (0.078–0.084)	0.048 (0.047–0.048)
	CRM (2.18 ± 0.18)	2.38 (2.37–2.39)	2.39 (2.34–2.44)	2.32 (2.24–2.41)	2.08 (2.06–2.09)	2.11 (2.08–2.14)	2.10 (2.00–2.21)	2.00 (1.93–2.08)	2.10 (2.08–2.12)
Se	Hep	6.76 (6.31–7.27)	7.12 (6.43–7.98)	7.53 (7.43–7.63)	5.46 (5.43–5.49)	6.13 (5.94–6.33)	6.30 (5.98–6.66)	6.48 (6.41–6.58)	4.84 (4.82–4.85)
	Claw	8.14 (7.60–8.76)	8.58 (7.74–9.62)	6.46 (6.37–6.55)	6.46 (6.42–6.50)	3.79 (3.67–3.92)	3.90 (3.70–4.12)	2.89 (2.85–2.93)	2.89 (2.88–2.90)
	CRM (1.62 ± 0.12)	1.41 (1.32–1.52)	1.49 (1.34–1.67)	1.57 (1.55–1.59)	1.07 (1.06–1.07)	1.33 (1.29–1.37)	1.37 (1.30–1.45)	1.41 (1.39–1.43)	1.05 (1.04–1.05)

Values are presented as concentration (95% confidence interval). The 95% confidence intervals quantify regression-based uncertainty derived from the corresponding calibration slope. Crab-tissue As estimates are presented for completeness only but were not for quantitative interpretation because of the poor calibration linearity. For each test matrix, calibration using the same matrix represents SA-IS, whereas calibration using a different biological matrix represents MMC. The CRM-based calibration curve generated in the Crab 1 analytical run was applied to Crab 2.

## Data Availability

The raw data supporting the conclusions of this article will be made available by the authors on request.

## Supplementary Materials

The following supporting information can be downloaded at: https://www.mdpi.com/article/10.3390/foods15213845/s1, Figure S1. Measured elemental concentrations under different calibration strategies; Table S1: Matrix effects (MEs) and associated statistical uncertainty for the investigated elements in the mussel certified reference material; Table S2: Concentrations (mg/kg, wet weight) of trace elements in brown crab (*Cancer pagurus*) hepatopancreas (Hep) and claw meat (Claw) and biases (%) of elemental determinations in certified reference material (CRM, mussel tissue ERM®-CE278k), using standard addition with internal standard (SA-IS), matrix-matched calibration (MMC) and external calibration (EC); Table S3: Parameters used in Equations (A1)–(A11).

## Appendix A

Equation (2) used for the element concentration calculation in the method of single-level standard addition with internal standard (SLSA-IS) was deduced as follows. All parameters used in Equations (A1)–(A11) are listed in Table S3.

The relationship between the analyte intensity and the concentration for x (the element of interest) and y (the IS, In) in a final sample solution (50 mL) could be expressed with Equations (A1) and (A2), respectively.

*I*_x_ = *k*_x_ × *C*_x_ + *b*_x_, (A1)

*I*_y_ = *k*_y_ × *C*_y_ + *b*_y_, (A2)

When the net intensity was used, Equations (A3) and (A4) were employed.

(A3) $$I_{x}^{\prime }=k_{x}\times C_{x},$$

(A4) $$I_{y}^{\prime }=k_{y}\times C_{y},$$

The ratios of the net signal intensity of x to that of y in the non-spiked and spiked final sample solution were expressed as Equations (A5) and (A6), respectively.

(A5) $$=\frac{I_{x}^{\prime }}{I_{y}^{\prime }}=\frac{k_{x}\times C_{x}}{k_{y}\times C_{y}},$$

(A6) $$R_{S}=\frac{I_{x(S)}^{\prime }}{I_{y(S)}^{\prime }}=\frac{k_{x}\times C_{x(S)}}{k_{y}\times C_{y(S)}},$$

When dividing Equation (A5) by (A6), Equation (A7) was obtained, where the *C*_y_ was seen as the same as *C*_y(S)_ since the addition of IS was equally carried out for all samples.

(A7) $$\frac{R}{R_{S}}=\frac{\frac{k_{x}\times C_{x}}{k_{y}\times C_{y}}}{\frac{k_{x}\times C_{x(S)}}{k_{y}\times C_{y(S)}}}=\frac{C_{x}}{C_{x(S)}},$$

For all samples, the concentrations of element x determined in 50 mL dilution were deducted from the concentration in the dry mass by Equations (A8) and (A9).

(A8) $$C_{x}=\frac{C_{x(\mathrm{dry})}\times m}{50\times 10^{-3}},$$

(A9) $$C_{x(S)}=C_{x}+C_{\mathrm{std}}=\frac{C_{x(\mathrm{dry})}\times m_{S}}{50\times 10^{-3}}+C_{\mathrm{std}},$$

Therefore, Equation (A7) could also be expressed as Equation (A10). The concentration of element x in the dry base was expressed as Equation (A11) by reorganising Equation (A10).

(A10) $$\frac{R}{R_{S}}=\frac{C_{x}}{C_{x(S)}}=\frac{\frac{C_{x(\mathrm{dry})}\times m}{50\times 10^{-3}}}{\frac{C_{x(\mathrm{dry})}\times m_{S}}{50\times 10^{-3}}+C_{\mathrm{std}}},$$

(A11) $$C_{x(\mathrm{dry})}=\frac{5\times 10^{-2}\times C_{\mathrm{std}}}{m\times \frac{R_{S}}{R}-m_{S}},$$
