# Supplementary material for: Calibration Strategies for Correcting Matrix Effects in ICP-MS Multi-Element Analysis Using a Certified Reference Material Followed by Application to Brown Crab (Cancer Pagurus) Tissues
Source: Foods. 2026 Oct 27;15(21):3845. doi: 10.3390/foods15213845 (PMC13762816; doi:10.3390/foods15213845)
Supplement: Supplementary file 1 [file foods-15-03845-s001.zip › foods-4566562-supplementary.pdf]

Supplementary Materials:

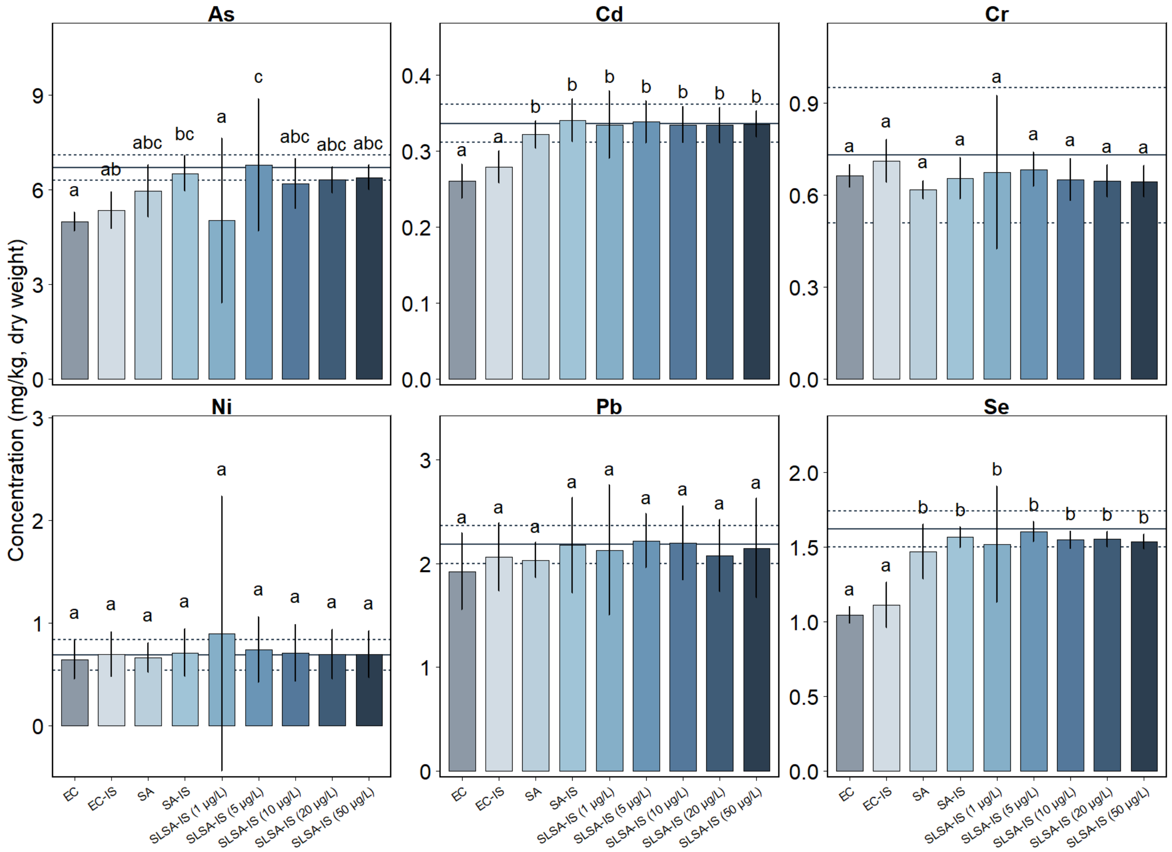

Figure S1. Measured elemental concentrations under different calibration strategies. Bars display the mean concentration (dry weight, mg/kg), $n = 3$. Error bars represent 95% confidence intervals of the mean. For each element, the horizontal solid line indicates the CRM certified value and the dashed lines denote the supplier reported uncertainty range. Within each facet, different letters indicate significant differences among calibration strategies within each element according to Tukey's HSD test ($p < 0.05$), following a significant Holm-adjusted omnibus ANOVA. Bar colours denote analytical methods: EC, external calibration; EC-IS, external calibration with internal standard; SA, standard addition; SA-IS, standard addition with internal standard; SLSA-IS (1–50 μg/L), single-level standard addition with internal standard at the indicated spike levels.

Table S1. Matrix effects (MEs) and associated statistical uncertainty for the investigated elements in the mussel certified reference material.

Element	Mean ME (%)	SD (%)	95% CI (%)	*p*-value
As	-15.12	6.22	[−30.56, 0.32]	0.052
Cd	-18.09	2.85	[−25.18, −11.00]	0.008
Cr	8.85	3.79	[−0.56, 18.26]	0.056

Ni	-2.07	2.75	[−8.90, 4.76]	0.322
Pb	-4.20	3.48	[−12.86, 4.45]	0.172
Se	-27.74	5.42	[−41.20, −14.27]	0.012

SD, standard deviation; CI, confidence intervals. p-values were obtained using two-sided one-sample t-test against 0%, n = 3.

Table S2. Concentrations (mg/kg, wet weight) of trace elements in brown crab (*Cancer pagurus*) hepatopancreas (Hep) and claw meat (Claw) and biases (%) of elemental determinations in certified reference material (CRM, mussel tissue ERM®-CE278k), using standard addition with internal standard (SA-IS), matrix-matched calibration (MMC) and external calibration (EC).

Element	Test tissue	Crab 1				Crab 2			
		Matrix source of calibration curve				Matrix source of calibration curve			
		Hep	Claw	CRM	EC	Hep	Claw	CRM	EC
As	Hep	10.45	16.93	18.71	15.58	8.92	9.32	12.20	9.80
	Claw	12.74	20.62	22.79	18.66	7.98	8.35	10.93	8.47
	CRM (6.7 ± 0.4)	−45.24%	−11.34%	−2.01%	−23.57%	−30.02%	−26.83%	−4.22%	−23.47%
Cd	Hep	3.35	3.11	3.07	2.57	1.95	1.82	1.68	1.49
	Claw	0.0036	0.0034	0.0033	0.0027	0.014	0.013	0.012	0.010
	CRM (0.336 ± 0.025)	11.53%	3.57%	2.06%	−20.07%	0.68%	−6.05%	−13.12%	−23.44%
Cr	Hep	0.031	0.031	0.034	0.038	0.023	0.022	0.027	0.025
	Claw	0.0071	0.0071	0.0077	0.0083	0.0092	0.0090	0.0117	0.0097
	CRM (0.73 ± 0.220)	−17.43%	−17.11%	−9.83%	−6.83%	−10.90%	−12.49%	−4.63%	−3.56%
Ni	Hep	0.16	0.16	0.17	0.17	0.15	0.15	0.16	0.15
	Claw	0.011	0.012	0.012	0.011	0.013	0.013	0.013	0.012
	CRM (0.69 ± 0.15)	7.59%	9.13%	14.89%	5.04%	9.45%	8.17%	13.46%	4.50%
Pb	Hep	0.041	0.041	0.040	0.038	0.079	0.079	0.085	0.080
	Claw	0.0091	0.0092	0.0089	0.0083	0.011	0.011	0.018	0.010

	CRM (2.18 ± 0.18)	9.03%	9.65%	6.35%	−4.73%	−3.30%	−3.55%	−8.13%	−3.86%
Se	Hep	2.33	2.46	2.60	1.88	1.88	1.93	1.99	1.49
	Claw	1.87	1.97	2.08	1.48	0.83	0.85	0.88	0.63
	CRM (1.62 ± 0.12)	−12.79 %	−8.07%	−2.82 %	−34.11 %	−17.76	−15.47 %	−12.87 %	−35.40 %

Crab-tissue As estimates are presented for completeness only but were not for quantitative interpretation because of the poor calibration linearity. For each test matrix, calibration using the same matrix represents SA-IS, whereas calibration using a different biological matrix represents MMC. The CRM-based calibration curve generated in the Crab 1 analytical run was applied to Crab 2.

Table S3. Parameters used in the Equations (A1)–(A11).

Parameter	Meaning	Parameter	Meaning
I_{x}	Signal intensity of the element of interest in the sample, cps.	I_{y}	Signal intensity of IS in the sample, cps.
k_{x}	Slope of the calibration curve for the element of interest.	k_{y}	Slope of the calibration curve for the IS.
C_{x}	Concentration of the element of interest in the sample dilution, μg/L.	C_{y}	Concentration of the IS in the sample dilution, μg/L.
b_{x}	Intercept of the calibration curve for the element of interest.	b_{y}	Intercept of the calibration curve for the IS.
I'_{x}	Net signal intensity of the element of interest in the sample, cps.	I'_{y}	Net signal intensity of the IS in the sample, cps.
R	Signal intensity ratio of the element of interest to the IS in the sample.	R_{S}	Signal intensity ratio of the element of interest to the IS in the spiked sample.
$C_{\mathrm{x(S)}}$	Concentration of the element of interest in the spiked sample dilution, μg/L.	$C_{\mathrm{y(S)}}$	Concentration of the IS in the spiked sample dilution, μg/L.
m	Mass of dry sample making non-spiked sample dilution, g.	m_{S}	Mass of dry sample making spiked sample dilution, g.
$C_{\mathrm{x(dry)}}$	Concentration of the element of interest in the dry sample, mg/kg.	C_{std}	concentration of the standard spike in the spiked sample dilution, μg/L.
